# Significance of a decrease in the proportion of detrusor muscle to bladder wall for non-invasive diagnosis of detrusor underactivity in men with lower urinary tract symptoms

**DOI:** 10.1038/s41598-022-09302-w

**Published:** 2022-03-28

**Authors:** Junghoon Lee, Sangjun Yoo, Min Chul Cho, Hyeon Jeong, Min Soo Choo, Hwancheol Son

**Affiliations:** Department of Urology, Seoul National University-Seoul Metropolitan Government Boramae Medical Center, Seoul National University College of Medicine, 20, Boramae-ro 5 gil, Dongjak-gu, Seoul, 07061 Korea

**Keywords:** Urology, Bladder

## Abstract

To investigate the significance of detrusor muscle thickness (DMT) to bladder wall thickness (BWT) ratio as a detrusor-sarcopenia and a consistently applicable factor for noninvasive diagnosis of detrusor underactivity (DU). We prospectively performed a urodynamic study of 100 male with medical refractory lower-urinary-tract-symptoms during 2017–2019. The DMT, BWT and DMT/BWT ratio were measured by ultrasonography every 50 mL during bladder filling, and were analyzed for non-invasive diagnosis of DU and prediction of prostate surgery outcome with questionnaire and the maximum-flow-rate. Of the 94 patients, DU was urodynamically diagnosed in 24 (25.5%). The DMT/BWT ratio was maintained in all patients until the 50% of the maximum cystometric capacity (MCC), and then rapidly decreased. At 20% of the MCC, the DMT/BWT ratio was significantly lower in the DU group (44.0 ± 4.9% vs. 49.4 ± 6.7%, *p* = 0.008). The DMT/BWT ratio of less than 47.5% at 20% of the MCC showed the ideal accuracy for diagnosing DU (AUC = 0.763), and was a predictor of failure at 12 months after prostate surgery (OR 8.78, *p* = 0.024). A DMT/BWT ratio of less than 47.5% at 20% of the MCC is a consistently applicable factor for non-invasive diagnosis of DU and could also be considered detrusor-sarcopenia.

## Introduction

Detrusor underactivity (DU) is a pathological term diagnosed low detrusor pressure or short detrusor contraction time through an invasive pressure-flow study test^1,2^. It is difficult to distinguish DU from other pathophysiologic conditions that cause lower urinary tract symptoms (LUTS) without an invasive pressure-flow study^2^. However, pressure-flow study is an invasive, expensive and time consuming test for patients. Recently ultrasonography has been proposed as an alternative to invasive pressure-flow study^3,4^. Recent study reported that ultrasound measurement of a thickened detrusor muscle could detect bladder outlet obstruction (BOO) better than the maximum urinary flow rate (Qmax) or even prostate volume^4^. An increase in bladder wall thickness (BWT) was also observed in women with DO on transvaginal ultrasonography^3^.

Sarcopenia is defined as low muscle strength and loss of muscle mass with aging^5^. There are similarities between sarcopenia and DU in terms of losing muscle strength. Indeed, one study reported a possible association between sarcopenia and impaired detrusor contractility^6^. The classic hypothesis of progression to DU explains that oxidative stress and/or progressive ischemia by chronic overload to the detrusor muscle lead to decompensatory underactivity and loss of detrusor muscle^7–9^. A decrease in the detrusor and bladder thickness may also be correlated with a decrease in bladder contractility^8,9^. Therefore, it is necessary to consider the concept of detrusor-specific sarcopenia because sarcopenia and DU are also similar in terms of losing muscle mass and strength. In addition to the absolute values of detrusor muscle thickness (DMT) and BWT, it is necessary to determine whether a decrease in the proportion of the detrusor muscle to the bladder wall is indicative of bladder contractility.

Some previous studies have attempted to explore methods of symptom-based or uroflowmetry parameters for the noninvasive diagnosis of pathologic DU^10–12^. Other studies also have reported that DMT or BWT on ultrasonography has an association with bladder contractility under limited bladder status^8,9^. However, the most controversy of DMT and BWT ultrasound measurement is that it is difficult to establish absolute reference ranges because of the changes of value according to the amount of bladder filling and each individual^13^. Some studies have also reported that detrusor hypertrophy is associated with bladder dysfunction^14,15^. Therefore, only measuring DMT or BWT is difficult to consistently apply to different individuals for DU diagnosis.

For the noninvasive diagnosis of DU, we would like to consider whether detrusor sarcopenia might be indicated by a decrease in the DMT to BWT ratio and investigate the significance of the DMT to BWT ratio as a consistent applicable method. And we want to investigate whether the DMT to BWT ratio is also related to the outcome of prostate surgery. Moreover, analyzing changes in the DMT, BWT and DMT to BWT ratio will help to further understand the physiology of the bladder, detrusor and DU.

## Materials and methods

### Design, setting, and participants

This study was approved by institutional review board of the Seoul National University-Seoul Metropolitan Government Boramae Medical Center (No. 10-2017-17). From December 2017 to October 2019, we prospectively recruited male patients aged 40 years or older who were scheduled for urodynamic evaluation for indications of refractory LUTS/benign prostatic hyperplasia (BPH) to medical treatments for more than 3 months. We excluded patients with medical conditions that could affect bladder function and/or structural deformation including neurological disorders of parkinsonism, stroke, multiple sclerosis, urethral stricture, bladder diverticulum, bladder stone, previous BPH or pelvic surgery, and prostate cancer. Patients who had complications of Clavien–Dindo grading 3 or higher after prostate surgery were also excluded.

The urodynamic studies (UDS) with pressure-flow study were conducted according to the International Continence Society guidelines for “Good Urodynamic Practice”^16^. Bladder outlet obstruction was determined for a bladder outlet obstruction index (BOOI) of 40 or more using detrusor pressure at Qmax (PdetQmax) and Qmax in a pressure-flow study. Urodynamic DU was defined as weak bladder contractility (bladder contractility index < 100) and bladder voiding efficiency < 90% without definite bladder outlet obstruction (BOOI < 40). BOOI and bladder contractility index was calculated according to the following equation, respectively: PdetQmax − 2 × Qmax; PdetQmax + 5 × Qmax^17,18^.

The laser surgery of BPH was considered for patients with bladder outlet obstruction who agreed to surgical treatment. At 3 and 12 months after surgery, surgical efficacy was determined by improvements in 3 domains related to surgical outcome: symptom domain, a reduction of 50% or more in the international prostate symptom score (IPSS); quality domain, an improvement of 3 points or more in the quality of life (QoL) score; and functional domain, an improvement of 5 mL/sec or more in Qmax^19,20^.

### Assessment of detrusor muscle thickness and bladder wall thickness

**Figure 1:**
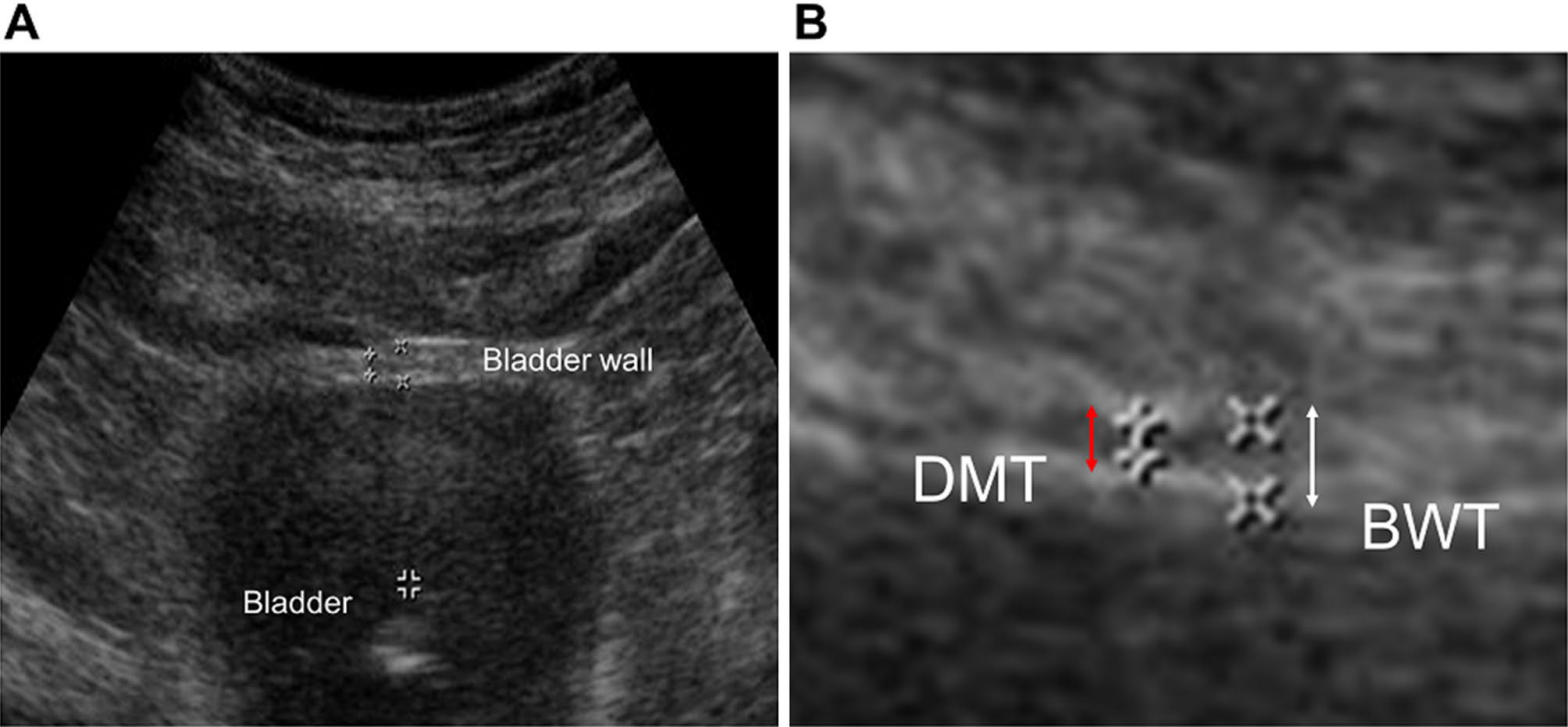
(**A**) Ultrasonography to measure the thickness of detrusor muscle and bladder wall, and (**B**) magnification to measure thickness. *DMT* detrusor muscle thickness, *BWT* bladder wall thickness.

Ultrasound measurements of the DMT and BWT were performed every 50 mL during filling in a cystometric study up to a bladder filling from empty to 500 mL or the maximum bladder capacity by one expert investigator blinded to the patient voiding status^21^. Because all parts of an individual bladder have been reported to have uniform thickness, the thickness of the bladder wall and detrusor muscle was measured at the anterior wall of the bladder with the patient in the supine position in this study. A 7.5 MHz linear array (HS60, Samsung Madison) was placed transversely 1 cm above the pubic crest, and sonographic images were obtained and stored digitally in the electronic imaging system. After magnification of the image approximately 10 times, the BWT and DMT were determined as the mean of thickness measurements at three different points separated by at least 1 cm. The BWT was measured as the width of the bladder wall including the two thin hyperechoic layers of mucosa, and the DMT measured the width of the hypoechoic space excluding hyperechoic layers (Fig. 1)^22^. The thickness ratio was calculated as (DMT/BWT × 100) %. Detrusor sarcopenia might be considered as a thickness ratio under the optimal cutoff value that predicted DU in receiver operating characteristic (ROC) analysis in this study.

### Statistical analysis

All data are presented as the median and interquartile range (IQR) or the mean and standard deviation. Student’s t test or the Mann–Whitney U test was used to compare continuous variables, and the chi-square test or Fisher’s exact test was used for categorical variables. Multiple logistic regression was used to evaluate the preoperative variables and determine which could be predictors of surgical outcome. The area under the ROC curve (AUC) was calculated to evaluate the predictive accuracy of the thickness ratio in diagnosing urodynamic DU. *p*-value < 0.05 was considered statistically significant. Statistical analyses were performed using IBM SPSS software v.26.

### Ethical approval

This study was approved by institutional review board of the Seoul National University-Seoul Metropolitan Government Medical Center (No. 10-2017-17).

### Consent to participate

Informed consent was obtained from all the participants. All study protocols were conducted in compliance with the principles of the Declaration of Helsinki guidelines.

## Results

A total of 100 screened male patients, 6 were excluded: three with stroke history, one with bladder diverticulum, one with Clavien–Dindo grade 3 complication and one with bladder stones. Of the 94 patients who underwent a UDS with refractory LUTS/BPH, the median age of the included patients was 71 (IQR 66–75) years, and the median prostate volume was 48 (IQR 39–73) mL (Table 1). A total of 24 (25.5%) were diagnosed with urodynamic DU. No clinical indicators could distinguish the DU from the non-DU patients.

**Table 1:** Patient baseline characteristics, International Prostate Symptom Score (IPSS) and urodynamic parameters.

	Total (n = 94)	DU (n = 24)	Non-DU (n = 70)	*p*
**Baseline, median (IQR)**				
Age, yr	71 (66–75)	73 (70.5–75.5)	71 (66–74)	0.056
BMI, kg/m^2^	24.0 (22.8–25.4)	23.3 (22.3–24.9)	24.1 (22.9–25.5)	0.356
DM, n (%)	27 (28.7%)	10 (41.7%)	17 (24.3%)	0.247
HTN, n (%)	50 (53.2%)	14 (58.3%)	36 (51.4%)	0.550
CVA, n (%)	6 (6.4%)	2 (8.3%)	4 (5.7%)	0.742
PSA, ng/ml	3.50 (1.43–6.62)	3.31 (1.62–5.67)	3.56 (1.41–7.36)	0.352
TRUS, mL	48 (39–73)	43 (35–47)	55 (40–83)	0.297
**Questionnaire**				
IPSS total	20 (12–29)	21 (11–25)	19 (12–29)	0.874
IPSS voiding	12 (6–17)	15 (6–17)	12 (7–17)	0.859
IPSS storage	8 (5–11)	7 (5–9)	8 (5–11)	0.488
OABSS	5 (4–8)	7 (4–9)	5 (4–8)	0.786
**Urodynamics, mean ± SD**				
Free Qmax, mL/s	8.9 ± 4.2	8.3 ± 4.1	9.1 ± 4.3	0.522
Free VV, mL	161.3 ± 93.9	143.6 ± 106.0	166.8 ± 90.4	0.423
Free PVR, mL	88.3 ± 88.1	93.7 ± 81.1	86.6 ± 91.1	0.789
First desire to void, mL	175.9 ± 76.9	191.4 ± 84.3	170.8 ± 74.7	0.375
Normal desire to void, mL	254.9 ± 99.7	255.5 ± 78.4	254.8 ± 106.6	0.979
Strong desire to void, mL	333.6 ± 100.4	349.5 ± 81.4	328.3 ± 106.3	0.349
MCC, mL	336.2 ± 97.9	349.5 ± 81.4	331.8 ± 103.3	0.548
Compliance, mL/cmH_2_O	50.9 ± 36.5	48.1 ± 39.3	51.9 ± 35.9	0.728
IDC ( +), n (%)	66 (70.2%)	18 (75.0%)	48 (68.6%)	0.745
IDC terminal, n (%)	31 (33.0%)	10 (41.7%)	21 (30.0%)	0.527
PdetQmax, cmH_2_O	59.3 ± 26.6	35.8 ± 6.7	67.3 ± 26.2	< 0.001
Opening Pressure, cmH_2_O	57.3 ± 33.3	34.2 ± 13.3	65.2 ± 34.5	< 0.001
BOOI	44.9 ± 28.0	22.7 ± 6.3	52.8 ± 28.3	< 0.001
BCI	92.3 ± 30.7	69.1 ± 21.0	100.1 ± 29.7	< 0.001
BVE	62.2 ± 25.2	52.9 ± 29.5	65.4 ± 23.1	0.102

*DU* detrusor underactivity, *TRUS* transrectal ultrasound, *IPSS* international prostate symptom score, *OABSS* overactive bladder symptom score, *Qmax* maximum flow rate, *VV* voided volume, *PVR* post-void residual, *MCC* maximum cystometric capacity, *IDC* involuntary detrusor contractions, *PdetQmax* detrusor pressure at Qmax, *BOOI* Bladder outlet obstruction index (PdetQmax − 2 × Qmax), *BCI* bladder contractility index (PdetQmax + 5 × Qmax), *BVE* bladder voiding efficiency (voided volume/total bladder capacity).

**Figure 2:**
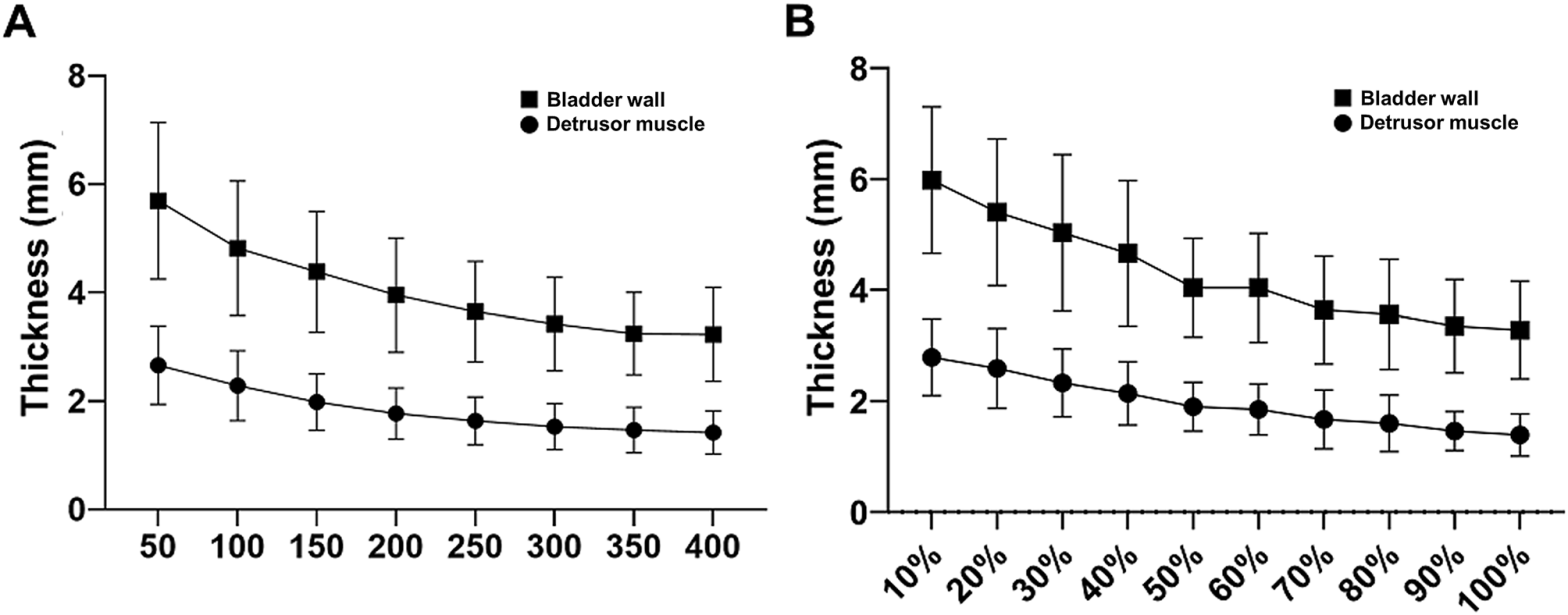
Changes in the BWT and DMT according to (**A**) the volume of bladder filling and (**B**) the ratio of bladder filling to the MCC. The DMT and BWT gradually decreased with bladder filling, and there was no significant difference (*p* > 0.05). *DMT* detrusor muscle thickness, *BWT* bladder wall thickness, *MCC* maximum cystometric capacity.

The DMT and BWT ranged from 0.6 to 4.4 mm and 1.6–9.1 mm, respectively. The both thicknesses were largest when the bladder was empty and gradually shrank as the bladder was filled (Fig. 2A). The mean BWT was 5.36 ± 1.29 mm at 20% of the maximum cystometric capacity (MCC), 4.04 ± 0.89 mm at 50% of the MCC, 3.54 ± 0.90 mm at 80% of the MCC, and 3.28 ± 0.85 mm at 100% of the MCC. Similarly, the mean DMT was 2.59 ± 0.72 mm at 20% of the MCC, 1.89 ± 0.44 mm at 50% of the MCC, 1.60 ± 0.50 mm at 80% of the MCC, and 1.39 ± 0.39 mm at 100% of the MCC (Fig. 2B). Relative to the initial thickness, the BWT decreased to 56.5% on average, and the DMT decreased to 54.1% at 100% of the MCC. The DMT and BWT according to each bladder filling volume did not show any significant difference between DU and non-DU patients.

**Figure 3:**
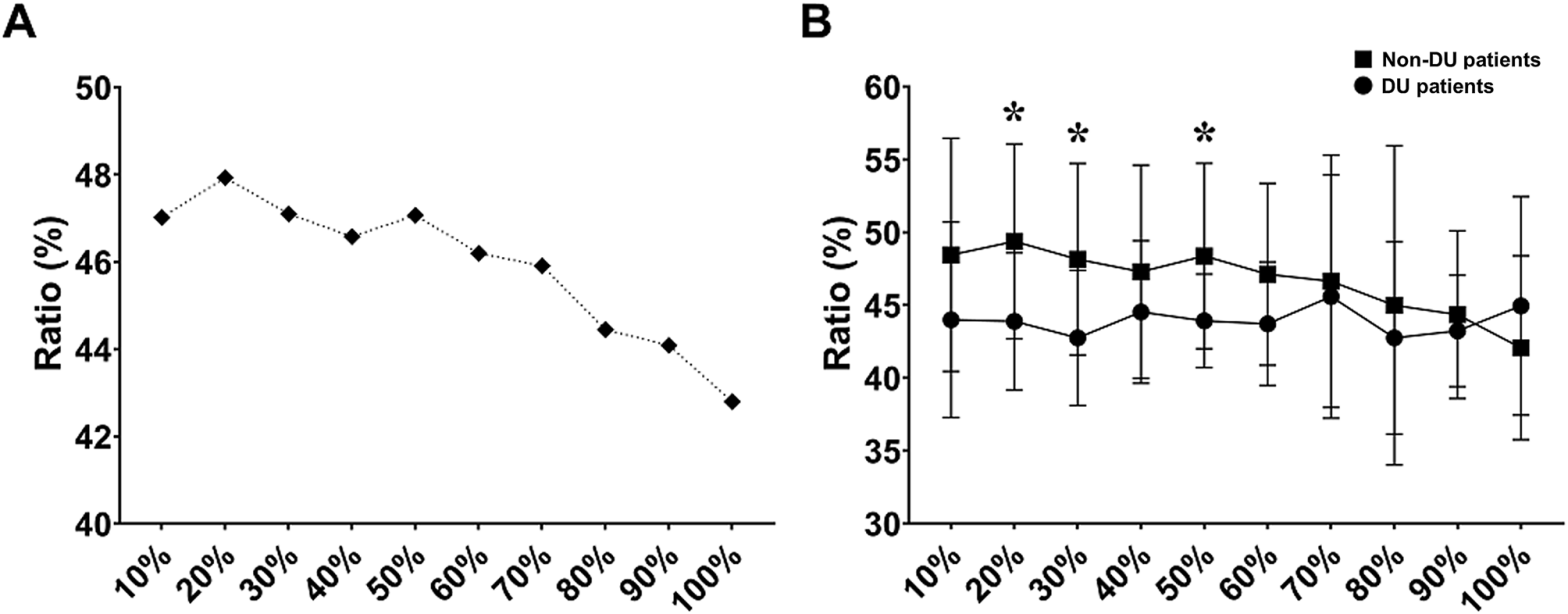
(**A**) The ratio of DMT to BWT for all patients and (**B**) the ratio of DMT to BWT for non-DU and DU patients, which changed according to the ratio of bladder filling to the MCC. The ratio of DMT to BWT was maintained until 50% of bladder filling to the MCC, and then decreased rapidly. Asterisks indicate significant differences in the ratios of DMT and BWT (*p* < 0.05). *DMT* detrusor muscle thickness, *BWT* bladder wall thickness, *MCC* maximum cystometric capacity, *DU* detrusor underactivity.

**Figure 4:**
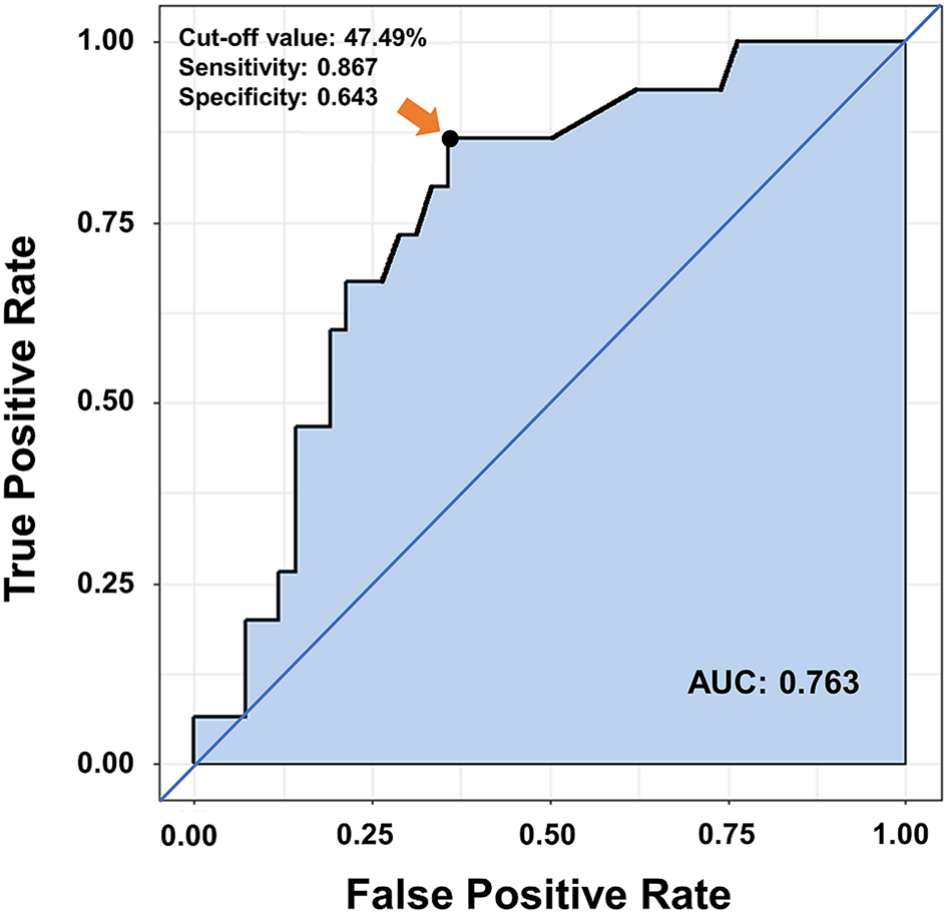
ROC curve presents the most ideal cutoff value of the ratio of DMT to BWT that can predict urodynamic detrusor underactivity when 20% of the MCC is filled (AUC = 0.763; the cutoff value: 47.5%). *ROC* receiver operating characteristics, *AUC* area under the ROC curve, *DMT* detrusor muscle thickness, *BWT* bladder wall thickness, *MCC* maximum cystometric capacity.

The thickness ratio was maintained at 47–48% until 50% of the MCC and then rapidly decreased to 42% at 100% of the MCC (Fig. 3A). The thickness ratio was significantly smaller in the DU group than in the non-DU group at 20% of the MCC (44.0 ± 4.9% vs. 49.4 ± 6.7%, *p* = 0.008). This difference was maintained when the bladder was filled to 30% and 50% of the MCC (42.8 ± 4.6 vs. 48.2 ± 6.6, *p* = 0.034; 43.9 ± 3.2 vs. 48.4 ± 6.4, *p* = 0.028), but ceased to be statistically significant when the bladder was further filled from 60 to 100% of the MCC (Fig. 3B). The AUC of the thickness ratio at 20% of the MCC was the highest with 0.763 (95% CI, 0.626–0.81) in detecting urodynamic DU (Fig. 4). The optimal cutoff value for predicting DU was 47.5% of the thickness ratio at 20% of the MCC.

Of the patients included in this study, 64 underwent prostate surgery. The overall success rates of BPH surgery were 56.1% and 48.8% at 3 months and 12 months, respectively. Detrusor sarcopenia was a risk factor for predicting surgical failure of postoperative 12 months (OR 8.78, *p* = 0.024) after adjusting for age, prostate volume, BOOI, and DU.

## Discussion

In this study, it was confirmed that the DMT/BWT ratio remained constant up to 50% of the MCC during bladder filling and then rapidly decreased. In patients with urodynamic DU, the thickness ratio was lower than that of non-DU from the beginning to half fullness. In addition, less than 47.5% of the thickness ratio at 20% of the MCC was a risk factor for a poor surgical outcome after laser prostate surgery. To the best of our knowledge, this is the first report using the thickness ratio for diagnosing urodynamic DU.

Sarcopenia patients experience not only a decrease in the size of the associated muscle but also a reduction in the quality of the muscle tissue over time^23^. The decreased quality of muscle is characterized by the infiltration of fat, an increase in fibrosis, changes in muscle metabolism, and degeneration of the neuromuscular junction^24^. Some studies have demonstrated that these changes are also induced in the detrusor muscles. The decompensated detrusor muscle in BOO had a reduced response to electrical stimulation, and its muscle fiber was replaced with fibrous connective tissue^2^. In studies on human tissue, a correlation was also observed between impaired contractility and degenerative structural changes with increased detrusor fibrosis^25^. Our study showed that a decreased proportion of the detrusor in the bladder wall was associated with DU.

It is known that the DMT and BWT decrease rapidly at the initial phase of bladder filling^21^. Previous animal studies have shown that the muscle layer is the most affected by volume changes^13^. Therefore, in patients with vulnerable detrusor muscle, the DMT could be more severely affected than other tissue layers, especially at the beginning of filling. To avoid these issues, some researchers recommend measuring the thicknesses after the bladder is filled to more than 250 mL^4^. However, since the bladder capacity is different for each individual, the effect of 250 mL filling on the bladder wall may vary. Other studies have also shown that the BWT constantly decreases even after exceeding 250 mL filling^26^. Ultrasonographic measurements of DMT and BWT as the bladder fills are more challenging because the wall thickness decreases. In our study, we confirmed that the thickness ratio remained relatively constant up to 50% of the MCC; thus, measuring the thickness ratio below 50% of the MCC would be more accurate and convenient.

Traditionally, the ultrasound measurements of the DMT and BWT have been investigated as possible noninvasive predictors of BOO or detrusor overactivity^3,4^. Some recent studies also have attempted to investigate the correlation between detrusor or bladder wall thickness and DU^8,9^. However, increases in DMT and BWT may not always increase bladder contractility. In the rabbit model for partial outlet obstruction, some rabbits showed severe bladder dysfunction despite hypertrophy of the detrusor smooth muscle^14,15^. In this study, the absolute value of the DMT or BWT did not show a significant difference between the DU and non-DU groups. On the other hand, reduction of the thickness ratio at 20%, 30% and 50% of the MCC was significantly associated with urodynamic DU. Our results suggest that the proportion of the detrusor muscle in the bladder wall may have a closer relationship with bladder contractility than the values of DMT and BWT. Since a thickness ratio of less than 47.5% at 20% of the MCC was associated with DU in this study, we suggest that it could be considered as the concept of detrusor sarcopenia.

DU is also a well-known risk factor for surgical failure^27^. However acceptable tools for noninvasively distinguishing BOO from DU have yet to be identified^8,28^. Huang et al.^29^ analyzed that increased BWT was associated with ineffective recovery of IPSS, QoL score and Qmax after surgery (OR 0.78, 95% CI 0.72–0.84). The DMT was analyzed as an independent predictor of surgical efficacy of IPSS, QoL score and Qmax (OR 2.05, *p* = 0.036; AUC 0.762, cutoff value 15 mm at the bladder volume of 250 mL)^30^. In this study, detrusor sarcopenia was identified as a noninvasive predictor of poor surgical outcome in Qmax and IPSS after 12 months of prostate surgery. Therefore, preoperative bladder ultrasonography for evaluation of the thickness ratio could be a potential, noninvasive method for identifying patients who require more detailed counseling regarding postoperative expectations.

This study also has several limitations. First, the number of DU patients was relatively small. This limitation might have biased the power of the statistical analysis. Second, the age of the two control groups was not matched in this study. However, it was difficult to conduct a large-scale and matched study because the previous studies were not sufficient and there were no established data. Because urodynamic studies are an invasive test, it is difficult to repeat additional tests including bladder filling for patients diagnosed with DU. Another limitation is that ultrasonography is not only operator-dependent, but also accumulates experience over time. For measuring wall thickness below 50% of the MCC, measuring DMT and BWT by ultrasonography is challenging. It will be necessary to secure several operators that can cross-validate and an appropriate control group through large-scale studies.

## Conclusions

This study showed that ultrasound-measured DMT to BWT ratio could serve as a noninvasive and consistently applicable diagnostic tool for predicting patients with urodynamic DU. To predict DU with noninvasive ultrasonography, we recommend measuring the DMT, BWT and thickness ratio when the bladder is filled with 20% of MCC. The optimal cutoff value of the thickness ratio for noninvasive diagnosis of DU was 47.5% at 20% of MCC. Therefore, the thickness ratio less than 47.5% at 20% of MCC could be considered a predictor of DU and a possible indicator of detrusor sarcopenia. DU was a risk factor that reduces the 12 months efficacy after prostate surgery.

## Data Availability

The datasets generated during and/or analyzed during the current study are available from the corresponding author on reasonable request.
